# Electrochemical analysis in charge-transfer science: The devil in the details

**DOI:** 10.1016/j.coelec.2021.100862

**Published:** 2021-10-19

**Authors:** Omar O’Mari, Valentine I. Vullev

**Affiliations:** 1 Department of Bioengineering, University of California, Riverside, CA 92521, USA; 2 Department of Chemistry, University of California, Riverside, CA 92521, USA; 3 Department of Biochemistry, University of California, Riverside, CA 92521, USA; 4 Materials Science and Engineering Program, University of California, Riverside, CA 92521, USA

**Keywords:** Charge transfer, Cyclic voltammetry, Reference electrode, Born solvation, Liquid junction, Effective radius

## Abstract

It is easy to carry out electrochemical analysis. It is demanding, however, to do it right, as inherent challenges, emerging from details in the data collection and the result interpretation, frequently present themselves. In pertinence to electron–donor–acceptor interactions, herein, we focus on voltammetrically obtained electrochemical potentials and their immense utility for extracting important characteristics of molecular analytes. Recommendations how to address key pending challenges, based on recent developments in electroanalysis and charge-transfer science, accompany the discussions on undesired impacts from irreversibility of oxidation and reduction, supporting electrolytes, choices of reference, liquid junctions, and ‘nonideality’ of molecular shapes. As the wide implications of charge transfer are indisputable, using the tools at our disposal for improving the reliability of electroanalysis is crucial for advancing modern science and engineering.

## Introduction

Heterogeneous charge transfer (CT) is the principal drive for electrochemical transformations. At the same time, electrochemical analysis is of utmost importance for evaluating the energetics of CT processes, such as electron transfer and hole transfer (HT). In particular, the electrochemical potentials of a donor and an acceptor are crucial for estimating the thermodynamic driving force (−Δ*G* ^(0)^) of CT between them [1].

Along with optical excitation energy, *ε*_00_, the reduction potentials of the acceptor$\left(E_{{\text{A}}^{y}|{\text{A}}^{\cdot (y-1)}}^{{}^{\left(0\right)}}\right)$ and of the oxidized donor $\left(E_{{\text{D}}^{\cdot (x+1)}|{\text{D}}^{x}}^{\left(0\right)}\right)$ define −Δ*G* ^(0)^ of photoinduced CT, as the Rehm—Weller equation implements [2,3]:

(1a) $$\Delta G_{\text{PCT}}^{\left(0\right)}(\in )=F\left(E_{{\text{D}}^{\cdot (x+1)}|{\text{D}}^{x}}^{\left(0\right)}\left(\in_{\text{D}}\right)-E_{{\text{A}}^{y}|{\text{A}}^{\cdot (y-1)}}^{\left(0\right)}\left(\in_{\text{A}}\right)\right)-\varepsilon_{00}+\Delta G_{S}\left(\in ,\in_{\text{D}},\in_{\text{A}}\right)+W(\in )$$

It is not to be confused with the Rehm—Weller equation that describes an empirical relationship between CT rate constants and driving forces that do not reveal Marcus inverted behavior [2,4].

The last term, *W*, accounts for the Coulombic interaction between the donor and the acceptor that, in this case, are non-radical species with initial charges *x* and *y*, respectively, separated at center-to-center distance *R*_DA_ [3]:

(1b) $$W(\in )=\frac{n(y-x-n)q_{e}^{2}}{4\pi \in_{0}\in R_{\text{DA}}}$$

The Born solvation term corrects for the interactions of the donor and the acceptor with the media, which for transferring *n* electrons is [3]:

(1c) $$\Delta G_{S}\left(\in ,\in_{\text{D}},\in_{\text{A}}\right)=\frac{nq_{e}^{2}}{8\pi \in_{0}}(\frac{2x+n}{r_{\text{D}}}\left(\frac{1}{\in }-\frac{1}{\in_{\text{D}}}\right)-\frac{2y-n}{r_{\text{A}}}\left(\frac{1}{\in }-\frac{1}{\in_{\text{A}}}\right))$$

Specifically, Δ*G*_*s*_ relates the reduction potentials of the donor and the acceptor, measured for solutions with dielectric constant ∈_D_ and ∈_A_, respectively, with the CT media with dielectric constant ∈ [3]. A decrease in the radii of the donor and the acceptor, *r*_D_ and *r*_A_, respectively, enhances the susceptibility of their reduction potentials to medium polarity.

The broad accessibility to cyclic voltammetry (CV), along with the ease of CV measurements, has made CV the principal experimental technique for obtaining electrochemical potentials [5–9]. As facile as CV and other voltammetry measurements are, inherent systematic errors in interpreting the potentials that electroanalytical techniques yield can prove quite misleading for CT analysis.

Herein, we introduce a concise overview of the electrochemical analysis for evaluating the energetics of CT processes. The presented recommendations, based on experimental and computational evaluations, allow for overcoming some of the challenges in the interpretations of electrochemical results.

### Electrochemical potentials and the thermodynamics of charge transfer

Koopmans’ theorem and its variations correlate measured $E_{{\text{B}}^{\cdot (q+1)}|B^{q}}^{\left(0\right)}$ with the energy level of the highest occupied molecular orbital (HOMO) of a species B^*q*^ with charge *q*; and $E_{{\text{B}}^{q}|{\text{B}}^{\cdot (q-1)}}^{\left(0\right)}$ with the level of its lowest unoccupied molecular orbital (LUMO) [10,11]. These correlations extend to utilizing ionization energy and electron affinity for estimating not only the levels of the frontier orbitals in molecular species, but also band energies of materials. Therefore, $F\left(E_{{\text{D}}^{\cdot (x+1)}|{\text{D}}^{x}}^{\left(0\right)}\left(\varepsilon_{\text{D}}\right)-E_{{\text{A}}^{y}|{\text{A}}^{\cdot (y-1)}}^{\left(0\right)}\left(\varepsilon_{\text{A}}\right)\right)$ in eq. (1a) correlates with the energy difference between the HOMO of the donor and the LUMO of the acceptor.

The zero-to-zero energy, ε_00_, or optical excitation energy, correlates with the difference between the LUMO and the HOMO of the photoexcited species. Upon excitation of the donor, therefore, $F\left(E_{{\text{D}}^{\cdot (x+1)}|{\text{D}}^{x}}^{\left(0\right)}\left(\in_{\text{D}}\right)-E_{{\text{A}}^{y}|{\text{A}}^{\cdot (y-1)}}^{\left(0\right)}\left(\in_{\text{A}}\right)\right)-\varepsilon_{00}$ represents the energy difference between the LUMO of the acceptor and the LUMO of the donor, and eq. (1a) yields the driving force of photoinduced ET, − Δ*G*_PET_^(0)^ [1,12,13]. For electrically excited acceptor, on the other hand, $F\left(E_{{\text{D}}^{\cdot (x+1)}|{\text{D}}^{x}}^{\left(0\right)}\left(\in_{\text{D}}\right)-E_{{\text{A}}^{y}|{\text{A}}^{\cdot (y-1)}}^{\left(0\right)}\left(\in_{\text{A}}\right)\right)-\varepsilon_{00}$ correlates with the energy difference between the HOMO of the accepter and the HOMO of the donor, and eq. (1a) estimates the driving force of photoinduced HT, −Δ*G*_PHT_^(0)^ [1,12,13].

Removing *ε*_00_ from eq. (1a) allows for evaluation of the driving force for CT between a donor and an acceptor, when both are in their ground electronic states [1]. It proves immensely important for estimating the driving forces of each of the discreet CT steps in long-range electron hopping from reduced species D^−^ to the LUMO of A:

(2a) $$\Delta G_{\text{ET}}^{\left(0\right)}(\in )=F\left(E_{\text{D}|{\text{D}}^{\cdot -}}^{\left(0\right)}\left(\in_{\text{D}}\right)-E_{\text{A}|{\text{A}}^{\cdot -}}^{\left(0\right)}\left(\in_{\text{A}}\right)\right)+\Delta G_{S}\left(\in ,\in_{\text{D}},\in_{\text{A}}\right)+W(\in )$$

and hole hopping from oxidized species A^+^ to the HOMO of D:

(2b) $$\Delta G_{\text{HT}}^{\left(0\right)}(\in )=F\left(E_{{\text{D}}^{\cdot +}|\text{D}}^{\left(0\right)}\left(\in_{\text{D}}\right)-E_{{\text{A}}^{\cdot +}|\text{A}}^{\left(0\right)}\left(\in_{\text{A}}\right)\right)+\Delta G_{S}\left(\in ,\in_{\text{D}},\in_{\text{A}}\right)+W(\in )$$

As conveniently illustrative as these MO ways of thinking are, the correlations between the electrochemical potentials and the energy levels of the frontier orbitals warrant a great deal of caution. Strictly speaking, electrochemical potentials represent energy differences between the reduced and oxidized states. Similarly, *ε*_00_ represents the energy of transitions between ground and excited states. That is, the electrochemical and spectroscopic measurements yield energy differences between different states rather than between different frontier orbitals. While this consideration unveils shortcomings of the Koopmans’ theorem, it actually validates the use of electrochemical potentials for analyzing the thermodynamics of CT as eqs. (1) and (2) implement.

## Estimating electrochemical potentials when samples “misbehave”

Standard electrochemical potentials, *E*^(0)^, as extensively obtained from CV measurements, provide key characteristics of the electronic properties of donors and acceptors. Using the average between the anodic and cathodic potentials, i.e., the half-wave potential, *E*^(1/2)^, has become an accepted representation of *E*^(0)^: Values of *E*^(1/2)^ are only attainable, however, when oxidation and reduction are reversible or at least, partially reversible [5,14].

**Figure 1:**
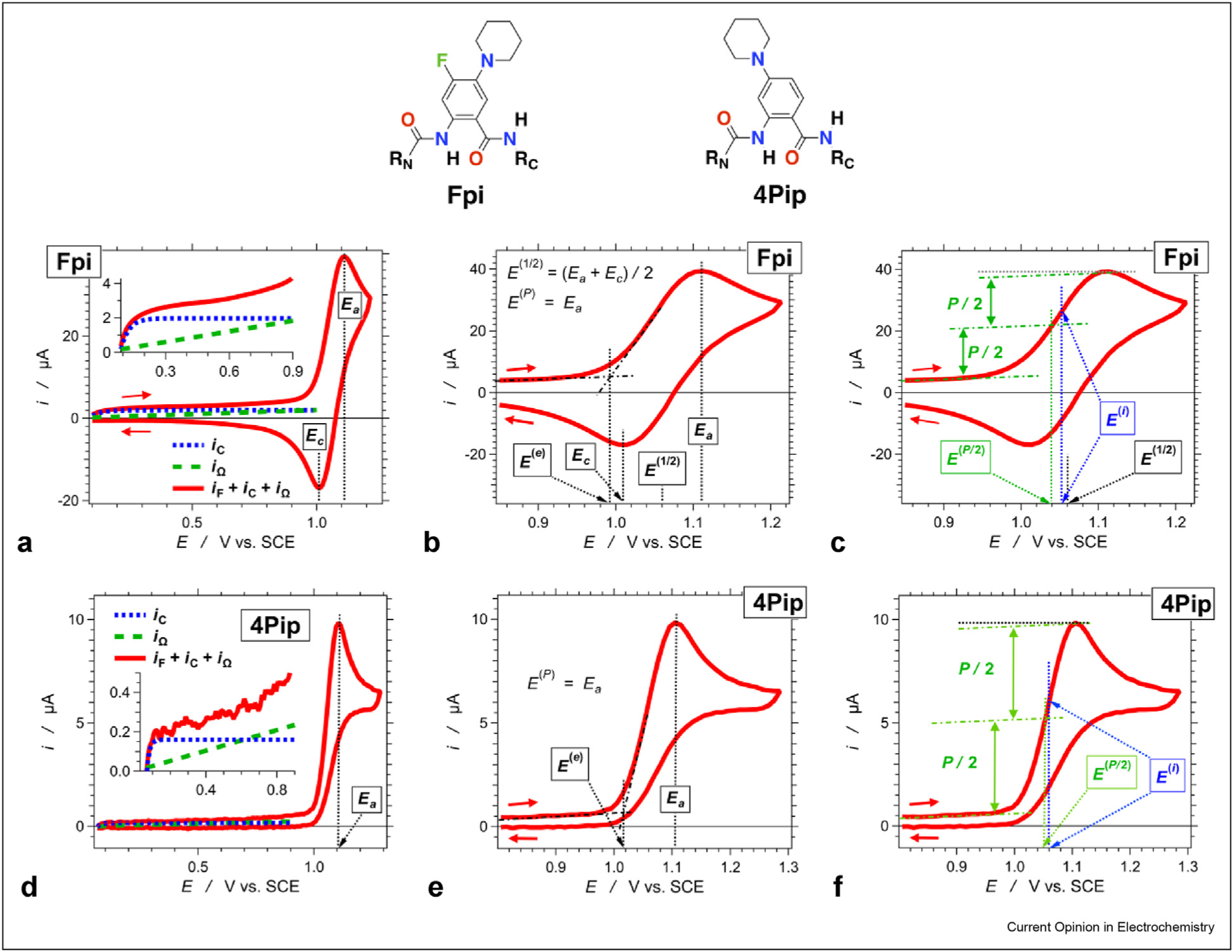
Cyclic voltammograms with the characteristic potentials designated. Cyclic voltammograms showing (**a–c**) reversible oxidation of 4-fluoro-5-(piperidin-*N*-yl)anthranilamide (Fpi) and (**d–f**) irreversible oxidation of 4-(piperidin-*N*-yl)anthranilamide (4Pip). Samples are dissolved in CH_2_Cl_2_ in the presence of 50 mM N(*n*-C_4_H_9_)_4_PF_6_, as reported in the study reported by Larsen-Clinton *et al*. [15]. The voltammograms illustrate the following: (**a,d**) the capacitance, *i*_C_, Ohmic, *i*_Ω_, and Faradaic, and *i*_F_, currents (**b,e**) the anodic, *E*_*a*_, cathodic, *E*_*c*_, and half-wave, *E*^(1/2)^, potentials, along with the peak, *E*^(*P*)^, and edge, *E*^(*e*)^, potentials; and (**c,f**) comparison of the inflection, *E*^(*i*)^ and half-peak potentials, *E*^(*P/*2)^, with *E*^(1/2)^. The values of *E*^(*i*)^ are obtained from the second derivatives of the voltammograms, that is, at the potentials where *∂*^2^*i*/*∂E* ^2^ = 0, whereas *dE*/*dt* = constant.

When irreversible behavior prevails, it is popular to report peak potentials, *E*^(*P*)^, or edge potentials, *E*^(*e*)^, as estimates of *E*^(0)^ (Figure 1a,b,d,e). As cyclic voltammograms of reversible processes reveal, however, *E*^(*P*)^ inherently overestimates *E*^(1/2)^ of oxidation and underestimates *E*^(1/2)^ of reduction (Figure 1b) [5]. Similarly, *E*^(*e*)^ underestimates *E*^(1/2)^ of oxidation, and overestimates *E*^(1/2)^ of reduction (Figure 1b) [5].

A few years ago, we introduced the utility of the potentials at the first inflection points (*E*^(*i*)^) on the anodic and cathodic waves of, respectively, irreversible oxidation and reduction, as estimates for *E*^(1/2)^ [16,17]. Actually, in pulse polarography, *E*^(1/2)^ is defined as the potential at the half-height currents, *i*^(1/2)^, of the sigmoid voltammograms (corrected for the Ohmic slopes), i.e., *E*^(1/2)^ = *E*(*i*^(1/2)^) where *i*^(1/2)^ = *i*_max_ − *i*_min_, and for reversible behavior, *E*^(1/2)^ = *E*(*∂*^2^*i*/*∂E* ^2^ = 0) = *E*^(*i*)^. Recent analysis shows that these inflection potentials, *E*^(*i*)^, indeed, offer the best representations of *E*^(1/2)^ (Figure 1c) [5]. The half-peak potentials, *E*^(*P*/2)^, represent another good estimator for *E*^(0)^ from irreversible voltammograms [6,18]. The estimation of *E*^(*P*/2)^ values, however, depends on the current/voltage baseline and the peak values. Hence, *E*^(*P*/2)^ cannot hold too well for complex voltammogramic waves when they appear broad with multiple peaks or when the capacitance and ohmic currents are comparable to the Faradaic signals (Figure 1a,d) [5]. Thus, *E*^(*i*)^ conduces to be a better representation of *E*^(1/2)^ than *E*^(*P*/2)^ (Figure 1c,f).

## To be or not to be … an electrolyte

The answer is ‘to be.’ Supporting electrolytes of ions, which are inactive within the electrochemical windows of the analyses, are essential for sufficiently high conductance of sample solutions in nonionic liquids. High sample resistance increases the Ohmic currents and affects the working electrode (WE) polarization, causing deviations of *E*^(1/2)^ from *E*^(0)^ (Figure 1a,d) [3,7].

**Figure 2:**
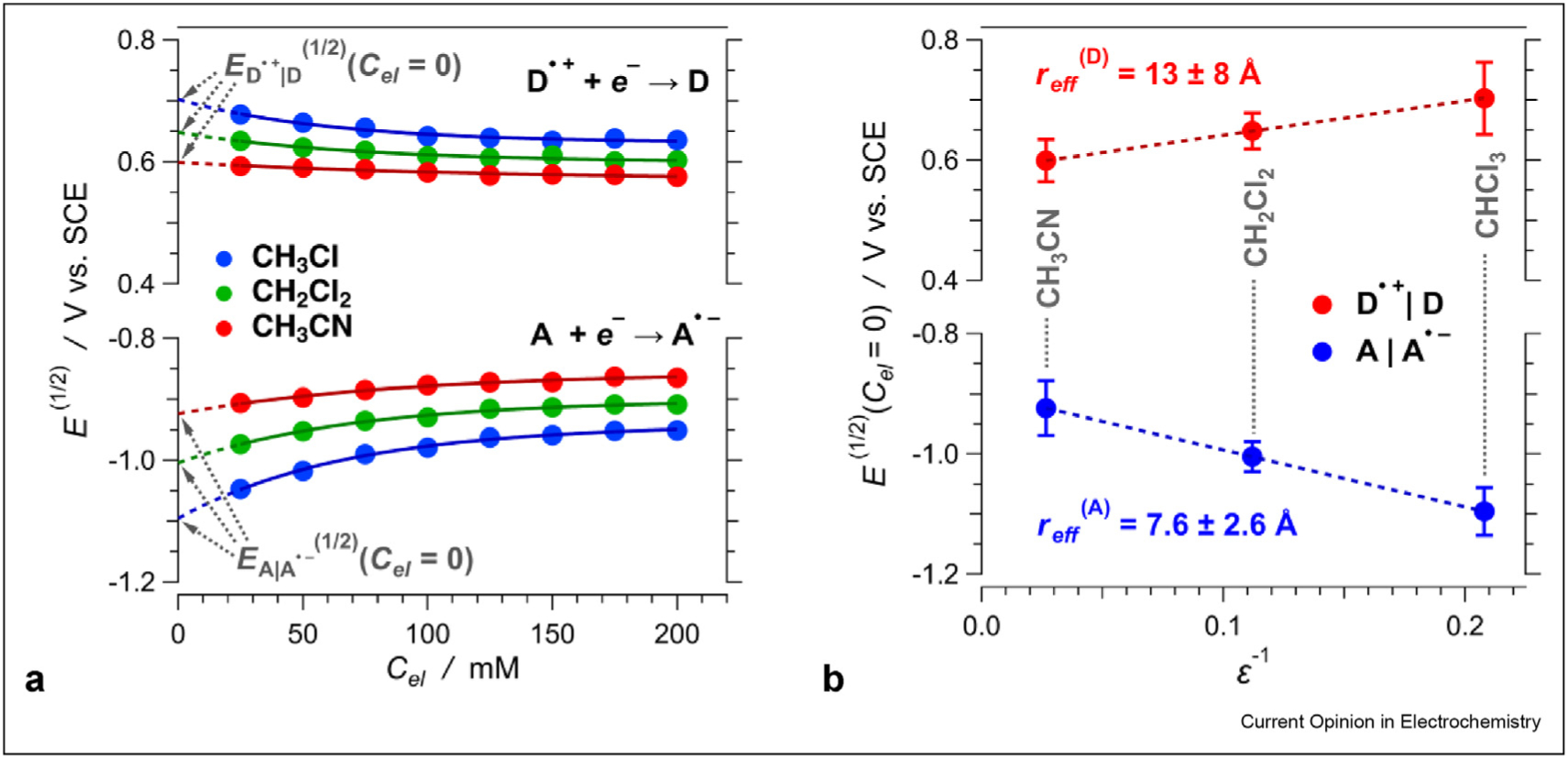
Dependence of the reduction potentials of an acceptor and an oxidized donor on medium polarity for *n* = 1 and *x* = *y* = 0 (eq. (1)). (**a**) Dependence of the reduction potentials on the concertation of the supporting electrolyte, *C*_*el*_, showing the extrapolation of the potentials for neat solvents, that is, at *C*_*el*_ = 0. (**b**) Dependence of the reduction potentials, extrapolated for *C*_*el*_ = 0, on the solvent polarity, expressed as the inverse static dielectric constant, along with the estimated effective radii, *r*_*eff*_, of the donor and the acceptor.

Supporting electrolytes affect ∈_D_ and ∈_A_, especially for solutions in low-polarity solvents and requires extra care for estimating the CT driving forces (eq. (1) and (2)) [3,19]. Therefore, we extrapolate the reduction potentials for neat solvents, i.e., *E*^(1/2)^ for *C*_*el*_ = 0, from the dependence of *E*^(1/2)^ on *C*_*el*_ (Figure 2a) [3,16,20–22]. This approach allows ∈_D_ and ∈_A_ to adopt well-defined published values for the used solvents, rather than the electrolyte solutions, improving the reliability of the thermodynamic CT analysis [23,24].

## What makes reference electrodes trustworthy?

On their own, values of potentials are not truly useful. Conversely, differences between potentials (measured under identical conditions) and potentials reported against reproducibly reliable references are crucially important for science and engineering [25].

Reliable reference electrodes (REs) provide the baseline for comparing results from different measurements. In setups with three and more electrodes [26–28], the voltage differences between REs and the WEs quantifies the potentials that drive the electrochemical transformations of interest. Therefore, a RE has to maintain a stable potential during measurements. The high impedance of REs keeps the currents through them negligibly minute and prevents detectable voltage drops. The counter electrodes serve as sinks for the current through WEs.

To ensure reproducibility, REs are usually compartmentalized heterogeneous systems connected with the electrochemical cells via liquid junctions and electrolyte bridges. Inherent physical and chemical characteristics of redox couples are fundamental for designing reliable REs. Redox couples of metal electrodes, M, and their cations ${\text{M}}^{z+}$, in electrolytes saturated with counterions, X^−^, where ${\text{MX}}_{z}$ has immensely low solubility, are an excellent choices for reliable REs. The presence of solid ${\text{MX}}_{z}$ in X^−^-saturates solutions ensures the constant activity of ${\text{M}}^{z+}$ in the liquid phase, $a_{M^{z+}}$, which depends on the ${\text{MX}}_{z}$ solubility product, $K_{\text{sp}}=a_{{\text{M}}^{z+}}a_{{\text{X}}^{-}}{}^{z}$. Maintaining a constant level of $a_{{\text{X}}^{-}}$ is key for reproducibly stable REs, the potentials of which depend on $K_{sp}\left({\text{MX}}_{z}\right)$ and the standard electrode potential of M, $E_{{\text{M}}^{z+}|\text{M}}^{\left(0\right)}$. Saturated calomel electrode (SCE) and silver/silver chloride (Ag/AgCl) electrode are the best example of such REs that have become standards for reporting electrochemical potentials vs. them [25].

Broadening of electrochemical applications places demands on shifting to organic-based references and decreasing the sizes of all electrodes. Encompassing a high-impedance connection via a single metal wire, pseudo-reference electrodes (PREs) have proven invaluable for miniaturizing the electrochemical setups. The potentials of PREs, however, strongly depend on their surrounding environment. It warrants careful calibration of PREs with internal standards, and reproduction of at least some of the measurements against a well-characterized RE.

For organic samples, ferrocenium/ferrocene, Fc^+^|Fc, pair is the most widely used standard. The immense stability of the ferrocenium ion ensures that ferrocene undergoes reversible or quasi reversible oxidation in a broad variety of electrolyte solutions at experimentally accessible potentials [3,5]. The immense utility of ferrocene for calibrating electrochemical setups has led to the use of Fc^+^|Fc as a reference. Reporting electrochemical potentials vs. Fc^+^|Fc, however, is fundamentally wrong. The Fc^+^ reduction potential strongly depends on the electrolyte composition [3]. That is, Fc^+^|Fc potential does not have the invariance of an RE. Therefore, while ferrocene is one of the best internal standards, reporting an electrochemical potential vs. Fc^+^|Fc indisputably warrants the disclosure of Fc^+^ reduction potential measured against an RE under the exact same conditions.

## The hidden menace of liquid junctions

Liquid junctions (LJs) and electrolyte bridges provide electrical contacts between solutions in different compartments of electrochemical setups, while preventing cross-contamination. Ion transport across an LJ, along with solvent differences, produces LJ electrical potentials (*E*_LJ_) [29,30]. The measured voltage difference between the WE and the RE, thus, encompasses the potential across the surface of the WE, needed for the analysis, along with the *E*_LJ_ values of the LJs between the two electrodes.

The ion diffusion across LJs, driven by concertation gradients, usually contributes only a few mV to *E*_LJ_, which is one-to-two orders of magnitude smaller than the contribution from the diffusion induced by differences in chemical potential [29]. Also, the contributions of the ion transport to *E*_LJ_ are inversely proportional to the ion charge [29]. Therefore, supporting electrolytes of multi-charged ions with a small propensity for transport through the LJs offers a means for decreasing the undesired *E*_LJ_.

The contributions of the solvent differences to *E*_LJ_ vary widely, e.g., 40 and 100 mV for H_2_O|CH_3_CN and H_2_O|DMF junctions, respectively [29]. Placing immiscible solvents across a junction can induce not only a huge potential drop, but also a complete shutdown of the electrical connection. Therefore, employing electrolyte bridges with two or more LJs while keeping solvent miscibility in mind, e.g., water|CH_3_CN|CH_2_Cl_2_, eliminates the enormous *E*_LJ_ that a single junction between immiscible solvents may produce, e.g., water|CH_2_Cl_2_.

## Size matters … in an inverse manner

While the donor and acceptor radii, *r*_D_ and *r*_A_, are key for CT analysis (eq. (1c)), most redox species are not spherical. Computed generalized Born radii, *r*_GB_, which account for the spatial distribution of the partial charges in redox species of any shape, offer an excellent representation of *r*_D_ and *r*_A_ [19,31]. Conversely, electrochemistry provides an experimental means for estimating effective radii, *r*_*eff*_, of redox moieties, also, regardless their shapes [16,19]. Both, *r*_GB_ and *r*_*eff*_, represent radii of spherical ions with homogeneously distributed charges that experience the same solvation energy as the analyzed non-spherical species [19].

**Figure 3:**
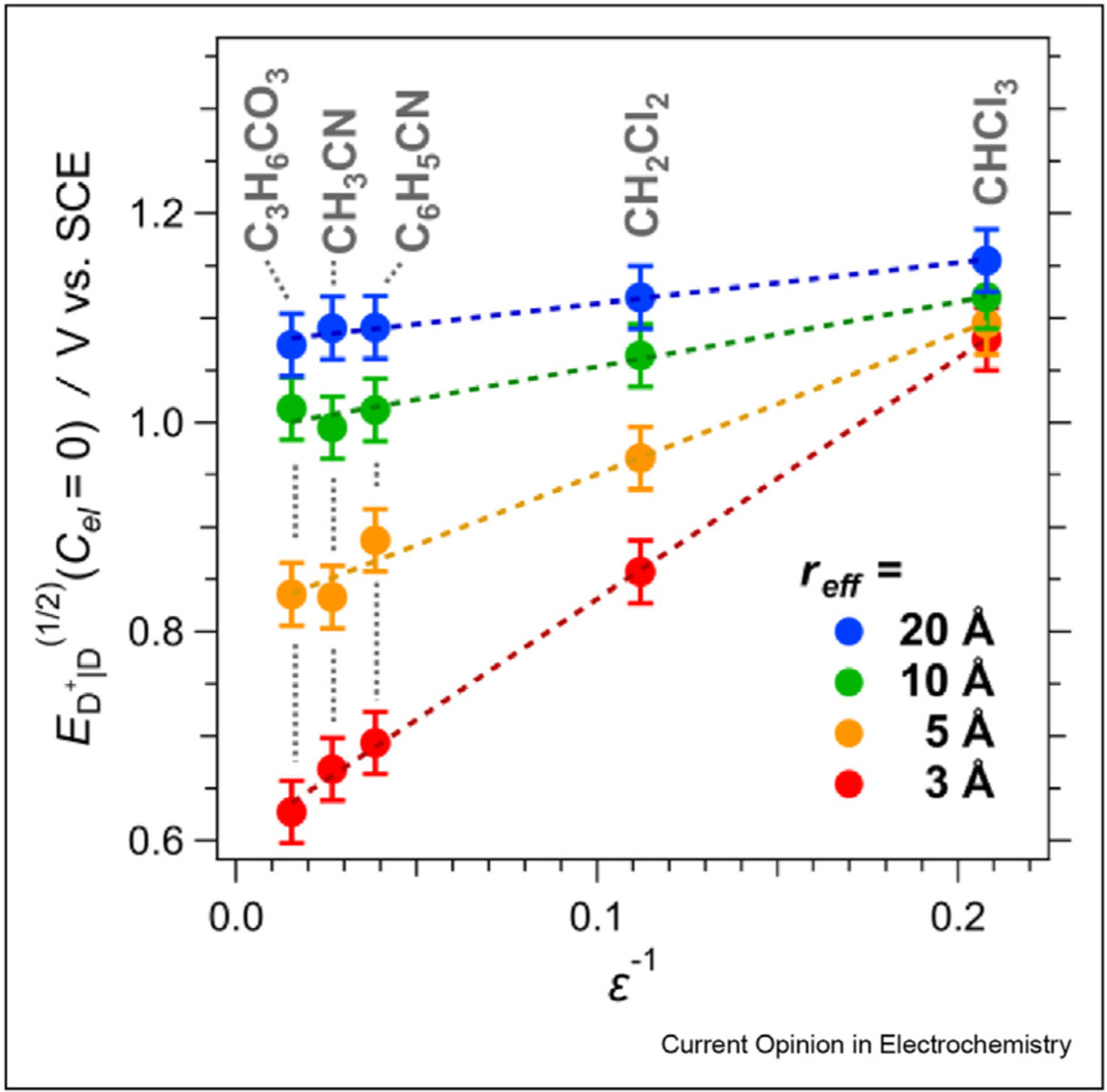
Polarity dependence of reduction potentials. Variations of the polarity dependence of the reduction potentials of oxidized donors on their effective radii, *r*_*eff*_, for *n* = 1 and *x* = 0 (eq. (1)).

The dependence of reduction potentials on medium polarity provides information about *r*_*eff*_. Specifically, *r*_*eff*_ is inversely proportional to the slopes of linear fits of *E*^(1/2)^ vs. ∈^−*1*^, i.e., *r*_*eff*_ = (8*π F* ∈_*0*_ slope)^−1^ (Figure 2b). This inverse relationship between potentials and the radii, however, compromises the reliability of *r*_*eff*_ estimates for large redox species. For *r*_*eff*_ exceeding 10Å, the difference between the reduction potentials for electrochemically feasible polar and non-polar solvents drops under about 0.1 V (Figure 3).

As important as *r*_A_ and *r*_D_ are, it is their inverse values, *r*_A_^−1^ and *r*_D_^−1^, that CT analysis implements (eq. (1c)). As we showed, when it is possible to measure the reduction potentials of the acceptor and the oxidized donor for the same media, i.e., ∈_A_ = ∈_*D*_ = ∈_*E*_, the separate values for *r*_A_ and *r*_D_ become redundant, as a rearrangement of eq. (1) reveals for *n* = 1 and *x* = *y* = 0 [12]:

(3a) $$\Delta G_{\text{PCT}}^{\left(0\right)}(\in )=F\Delta E^{\left(0\right)}(\in )-\varepsilon_{00}+W(\in )$$

(3b) $$\Delta E^{\left(0\right)}(\in )-\Delta E^{\left(0\right)}\left(\in_{\text{E}}\right)=\frac{q_{e}^{2}}{8\pi F\in_{0}}S_{1/r}\left(\frac{1}{\in }-\frac{1}{\in_{\text{E}}}\right)$$

where $\Delta E^{\left(0\right)}\left(\in_{\text{X}}\right)=\left(E_{{\text{D}}^{\cdot +}|\text{D}}^{\left(0\right)}(\in \text{X})-E_{\text{A}|{\text{A}}^{\cdot -}}^{\left(0\right)}\left(\in_{\text{X}}\right)\right)$ and $S_{1/r}=r_{\text{A}}^{-1}+r_{\text{D}}^{-1}$.

**Figure 4:**
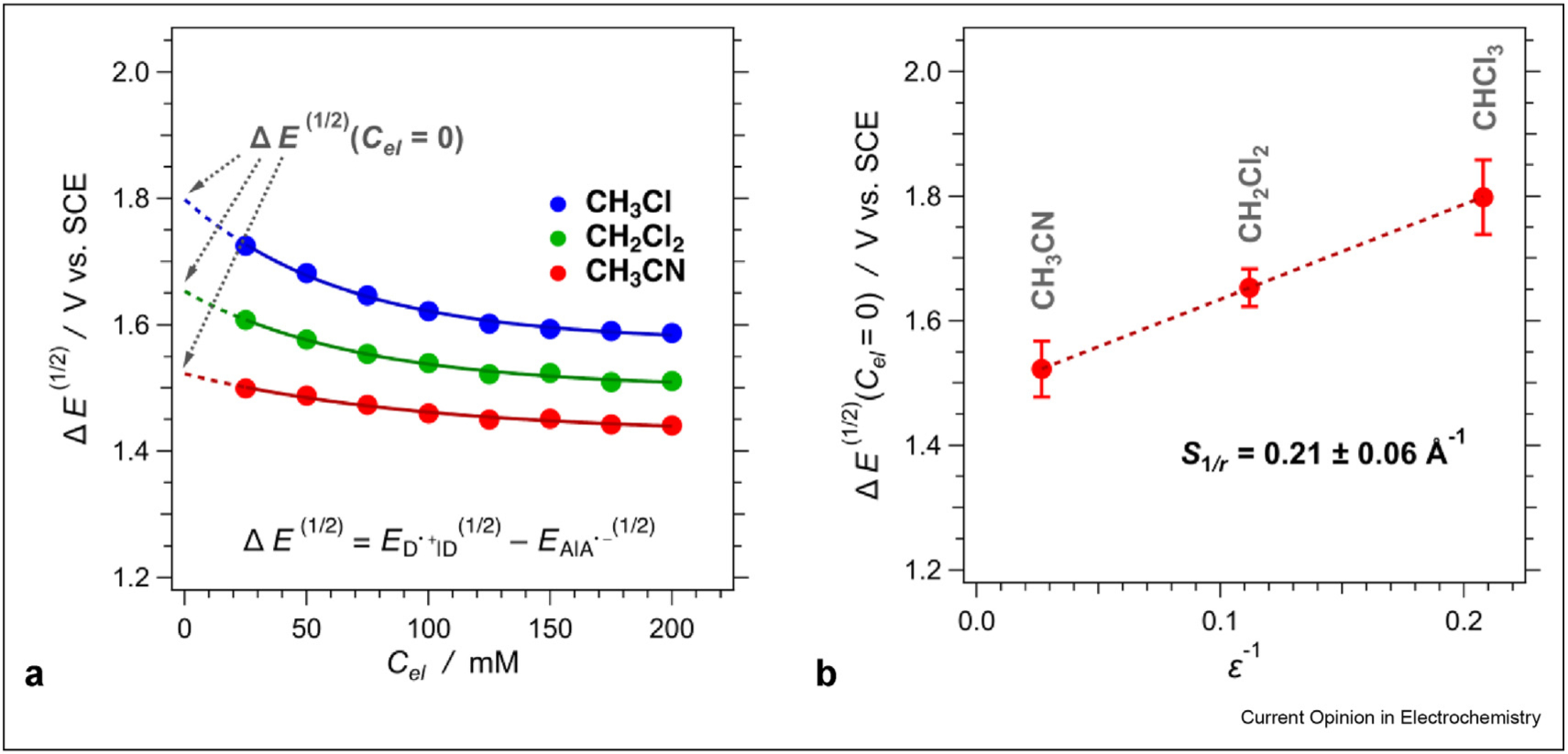
Polarity dependence of the potential differences for the donor-acceptor case presented in Figure 2.(**a**) Dependence of the potential differences on the concertation of the supporting electrolyte, *C*_*el*_, showing the extrapolations for neat solvents, that is, for *C*_*el*_ = 0. (**b**) Dependence of the potential differences, extrapolated for *C*_*el*_ = 0, on the solvent polarity, expressed as the inverse static dielectric constant, along with the estimated sum of inverse radii, *S*_1*/r*_ (eq. (3b)).

The polarity dependence of Δ*Е*^(1/2)^ produces the sum of inverse radii, *S*_1/*r*_ (Figure 4). Not only *S*_1/*r*_ is essential for implementing such simplified driving-force calculations (eq. (3)), but also employing $E_{{\text{D}}^{\cdot +}|\text{D}}^{\left(1/2\right)}$ and $E_{\text{A}|{\text{A}}^{\cdot -}}^{\left(1/2\right)}$ from measurements, using the same electrochemical setup with the same electrolyte solutions, eliminates from Δ*Е*^(1/2)^ the systematic errors from the liquid-junction potentials. In addition to improving the precision of obtaining *S*_1/*r*_, in comparison with that for attaining *r*_D_ and *r*_A_ for large species, the implementation of *S*_1/*r*_ extends beyond the CT thermodynamics. Outer-sphere, or medium, reorganization energy, *λ*_*m*_, is directly proportional to *S*_1/*r*_ [12]:

(4) $$\lambda_{m}=\gamma \frac{n^{2}q_{e}^{2}}{8\pi F\in_{0}}\left(\frac{1}{2}S_{1/r}-\frac{1}{R_{\text{DA}}}\right)$$

The calculations of *λ*_*m*_ employ a model of Born solvation originating from orientational and nuclear/vibrational polarization, as implemented by the Pekar factor, $\gamma =n_{m}^{-2}-\in_{m}^{-1}$ [1,13,23]. Therefore, *S*_1/*r*_ is an excellent representation of the sum of the inverse radii in eq. (4). After all, a Born-solvation analysis of electrochemical potentials produces *S*_1/*r*_ (Figure 4b).

## Conclusions

As an intricate part of charge-transfer science, the importance of electrochemical analysis cannot be overstated. Energy science, photoredox- and electro-catalysis, biomedical sensor development, environmental engineering and (opts)electronics are some of the areas that are strongly contingent on CT, and place demands for high-fidelity electrochemistry. Without losing sight of the big picture, therefore, paying attention to details, when gathering, implementing, and interpreting electrochemical results, is essential for advancing this broad range of areas of science and engineering.

## Abbreviations

*C_el_*: Concentration of supporting electrolyte
CT: Charge transfer
CV: Cyclic voltammetry
ε_00_: Zero-to-zero energy
*E^(0)^*: Standard electrochemical potential
*E^(1/2)^*: Half-wave potential
*E^(e)^*: Edge potential
*E^(i)^*: Infliction point potential
*E^(P/2)^*: Half peak potential
*E^(P)^*: Peak potential
*E_a_*: Anodic peak potential
*E_c_*: Cathodic peak potential
*E*_LJ_: Liquid junction electrical potential
ET: Electron transfer
*F*: Faraday constant
γ: Pekar factor
*G_S_*: Born solvation energy
HOMO: Highest occupied molecular orbital
HT: Hole transfer
*K*_sp_: Solubility product
*λ_m_*: Reorganization energy
LE: Locally excited
LJ: Liquid junction
LUMO: Lowest unoccupied molecular orbital
MO: Molecular orbital
*n*: Number of transferred electrons
*n_m_*: Refractive index of medium *m*
PCT: Photoinduced charge transfer
PRE: Pseudo-reference electrode
*q_e_*: Elementary charge
*r*_A_: Radius of acceptor
RE: Reference electrode
*r_eff_*: Effective radius
*r*_D_: Radius of donor
*r*_GB_: Generalized Born radius
*S*_1/*r*_: Sum of the inverse radii of an electron donor and an acceptor
SCE: Saturated calomel electrode
WE: Working electrode

## Abbreviations

∈_0_: Vacuum permittivity
∈: Relative static dielectric constants
∈_E_: Dielectric constantof media for electrochemical measurements
∈_A_: Dielectric constantof the solvent for measuring the potential of an electron acceptor
∈_D_: Dielectric constantof the solventfor measuring the potential of an electron donor
∈_X_: Dielectric constantof medium X
∈_*m*_: Dielectric constantof medium *m*
